# The use of text messages as an alternative invitation method for breast cancer screening: A randomized controlled trial (M-TICS study)

**DOI:** 10.1371/journal.pone.0306720

**Published:** 2024-08-29

**Authors:** Nuria Vives, Carmen Vidal, Ena Niño de Guzman, Albert Farre, Jon Aritz Panera, Gemma Binefa, Montse Garcia, on behalf of the M-TICS research group

**Affiliations:** 1 Cancer Screening Unit, Catalan Institute of Oncology, Hospitalet de Llobregat, Barcelona, Spain; 2 Early Detection of Cancer Research Group, EPIBELL Program, Bellvitge Biomedical Research Institute, L’Hospitalet de Llobregat, Barcelona, Spain; 3 Consortium for Biomedical Research in Epidemiology and Public Health (CIBEResp), Madrid, Spain; 4 School of Health Sciences, University of Dundee, Dundee, United Kingdom; Local Health Authority Caserta: Azienda Sanitaria Locale Caserta, ITALY

## Abstract

This study aimed to determine whether a text message is as good as a postal letter as an invitation method for previous screenees in a breast cancer screening program, considering a non-inferiority margin of -2 percent points on participation rate. A non-inferiority randomized control trial was conducted. Women in the intervention group (n = 5,362) were invited by text message, and women in the control group (n = 5,482) were invited by letter, which is the standard invitation procedure of the program. In both groups, the invitation included a fixed appointment for mammography and a text message reminder 96 hours before the appointment. The primary outcome was screening participation rate (completing mammography within 12 weeks of invitation). Secondary outcomes included mammography attendance to initial or rescheduled appointments and cancellation rate. The intention-to-treat analysis showed a participation rate of 87.3% and 86.6% in the control and intervention groups, respectively. The difference in participation rate was -0.7 percentage points (95% confidence interval [CI], -1.8 to ∞), indicating non-inferiority of text messages compared to letter invitations. The per-protocol analysis showed similar results. Attendance at the initial appointment was higher in women who received the text message invitation compared to those in the control group (P<0.002). Women who received the invitation by letter canceled more the initial appointment scheduled compared to the text message group (21.1% and 15.1%, P<0.007). In conclusion, we found that a text message invitation for women who had previously participated in breast cancer screening was not inferior to the standard letter. This randomized controlled trial provides valuable insights into the use of alternative invitation methods for population-based cancer screening programs. However, further research is needed to determine the best timing and frequency of text messages for better outcomes and identify strategies for facilitating rescheduling or cancellation.

**Trial Registration**: Clinicaltrials.gov NCT04343950, (04/09/2020).

## Introduction

Breast cancer (BC) screening is strongly recommended as it reduces cancer mortality despite some potentially harmful effects, such as overdiagnosis [1, 2]. The population-based BC screening program in Catalonia (Spain) invites women aged 50 to 69 for biennial mammography [3]. The health survey of Catalonia conducted in 2022 found that 86% of women of screening age have undergone a mammogram in the last two years, either through public or private healthcare services [4]. However, participation in the population-based BC screening program is about 65%. Furthermore, adherence among the participants in the program is above 85%, which is in line with European standards [5, 6].

Cancer screening programs have traditionally used postal letters as the main communication channel with their target population. However, alternative invitation methods have been proposed to increase the uptake of cancer screening programs [7, 8] and facilitate women to reschedule appointments in case they may not be able to attend the visit originally planned [9]. One method that has been widely recommended is the use of text messaging due to its acceptability, low cost, ubiquity, and capacity for personalization [10]. A previous systematic review included five studies evaluating text message reminders and showed a moderate increase in BC screening participation (4.5% to 15%) [11]. Furthermore, a quasi-experimental study in a BC screening program in Catalonia showed that text message reminders were acceptable [12]. As a result, the European Commission Initiative for Breast Cancer now recommends their implementation [13].

There is notably less focus on managing cancellations and/or rescheduling appointments using text message reminders. Cancellation of appointments may be considered a desirable outcome, especially when the cancellation occurs in time for the appointment to be reallocated to another individual. Rescheduling appointments may also be a desirable outcome as the individual benefit of the screening increases with the number of screens in which the women participated [14].

Text messages may be limited by their content and may restrict their use as an invitation method for initial screening participation, as they should receive detailed information on the benefits and harms of screening to make an informed decision about participation [15]. Previous participants could be an ideal subgroup to test alternative invitation channels as they have already decided to participate in the cancer screening program. However, since successive participation in breast cancer screening is high, the margin for improvement is limited. It is important to understand that women may sometimes forget or find it inconvenient to attend their scheduled appointments. In such cases, reminders that encourage cancellation of unwanted appointments and rescheduling to a more convenient time may be all that is warranted in breast cancer screening programs that already have a high attendance rate [16, 17]. To the best of our knowledge, no studies have been conducted to assess the effectiveness of using it as an invitation screening method.

A modest reduction in efficacy (participation rate) may be acceptable as a tradeoff for the secondary benefits of the text message invitation in terms of sustainability and cost reduction. As an additional benefit, text messages could improve cancellation rates and/or rescheduling appointments.

This randomized control trial aimed to determine whether text messages are non-inferior to (as good as) the standard postal letter as an alternative invitation method for mammographic screening in previously participating women in a BC screening program. A noninferiority margin of -2 percent points on the participation rate was considered.

## Methods

### Design

We conducted a noninferiority randomized controlled trial from 14 September 2021 to 26 April 2022. We compared a text message invitation method with the standard invitation procedure (a letter sent by mail) in a population-based BC screening program. This trial is part of the M-TICS Study, with the protocol previously published [18] according to the SPIRIT statement. The study received ethical approval from the Ethics Committee of the Bellvitge University Hospital (PR042/20), which waived the requirement to obtain the participant’s signature as part of the consent process, as the intervention was a minor variation on the invitation practice.

### Setting

The Catalan Institute of Oncology hub, as part of the BC screening program in Catalonia (Spain), manages a biennial population-based screening program for BC in the Southern Metropolitan Area of Barcelona and covers a target population of 191,957 women aged 50–69 (1 January 2022). The hub identifies women who are due for screening from the Central Register of Insured Persons of the Catalan Health Service. A centralized appointment scheduling system to allocate all eligible women within a two-year interval is used. Eligible women receive an invitation letter with a timed appointment to perform mammography at their referral radiology unit. A leaflet with information to decide whether to be screened is enclosed for women invited for the first time. If an invitation letter is returned to our hub due to an incorrect mailing address, we verify its current and correct status. If we find that the address is incorrect, we send a new appointment invitation to the updated address.

### Participants and randomization

Eligible participants were women who had previously participated in our program (in the previous 30 months) and were scheduled to receive their next invitation for BC screening during the recruitment period of the trial. We designed an application using JavaScript’s built-in Math.random function to select and randomize eligible women in a 1:1 ratio to the intervention or control group. From 14 September 2021 to 15 October 2021 and from 17 January 2022 until 26 April 2022, eligible women were randomized to the intervention daily until the target sample size was achieved. Women without a registered mobile phone were excluded. No blinding was considered at any step. However, the endpoint of this study did not require subjective judgment.

### Intervention

Women randomly assigned to the control group received the standard procedure: an invitation letter with a timed appointment for mammography. Women randomly assigned to the intervention group received a text message invitation with a timed appointment for mammography. Text messages were bidirectional (enabling two-way messaging) and fully automated delivery through a platform. The screening hub staff managed the incoming individual responses. The initial of the first name and the entire last name were part of the text message. A link to obtain more information about the appointment was also provided. An invitation letter was sent to the women for whom text messages failed to be delivered. In both groups, as part of the standard screening invitation process, an automated text message reminder was sent 96 hours before the timed appointment (S1 File).

### Outcomes and baseline variables

The primary outcome was the screening participation rate, defined as women performing screening mammography within 12 weeks of the invitation. Secondary outcomes measured included attendance rates for the initial or rescheduled appointments and cancellation rates among non-attenders. Baseline variables were age at invitation and tertiles of deprivation score index (DS), based on the individual’s Catalan primary healthcare referral area [19].

### Statistical analysis

The sample size was estimated at 10,908 women, considering 15% of women without mobile phone numbers, 10% of wrong mobile phone numbers recorded, and a 2-percentage-point non-inferiority margin in the absolute risk difference scale, with a one-sided alpha level of 0.05, and 90% power. The expected participation among women with regular successive screening was estimated at 86%. The 2-percentage-point non-inferiority margin was the difference between the maximum and minimum participation rates in the last ten years in our screening program.

Descriptive statistics were computed as means and standard deviation (SD) for continuous variables and as numbers and percentages for categorical variables. Baseline characteristics and secondary outcomes between the study groups were compared using the Student t-test for continuous data and Chi-square tests for categorical data. The null hypothesis was the inferiority of the participation for textmessage invitations by at least 2% of the participation for letter invitations at 12 weeks. The non-inferiority margin was compared with the inferior limit of the one-sided 95% confidence interval (CI) of the difference in participation. The intention-to-treat analysis comprised all women initially randomized to either the intervention or control groups. In contrast, the per-protocol analysis included only women who received the assigned invitation. Exploratory subgroup analyses were performed by age and DS index tertile. For subgroup analysis of participation, point estimates and 95% CIs were considered instead of the non-inferiority margin of 2 percentage points and were adjusted for multiple testing. All the analyses were performed using STATA version 18.0 (Stata Corp LP, College Station, Texas).

### Interim analysis

In December 2021, the interim analysis including 3,952 participants (intention to treat population) showed no difference in participation rate between the text message group and control group (87.3% vs. 88.5%, respectively, P = 0.257). As the participation rate in the text message group was slightly lower, although not statistically significant, we added a second text message together with the text message scheduled appointment to inform women that the text message replaced the usual invitation letter (S1 File and S1 Table).

## Results

### Participants

**Fig 1:**
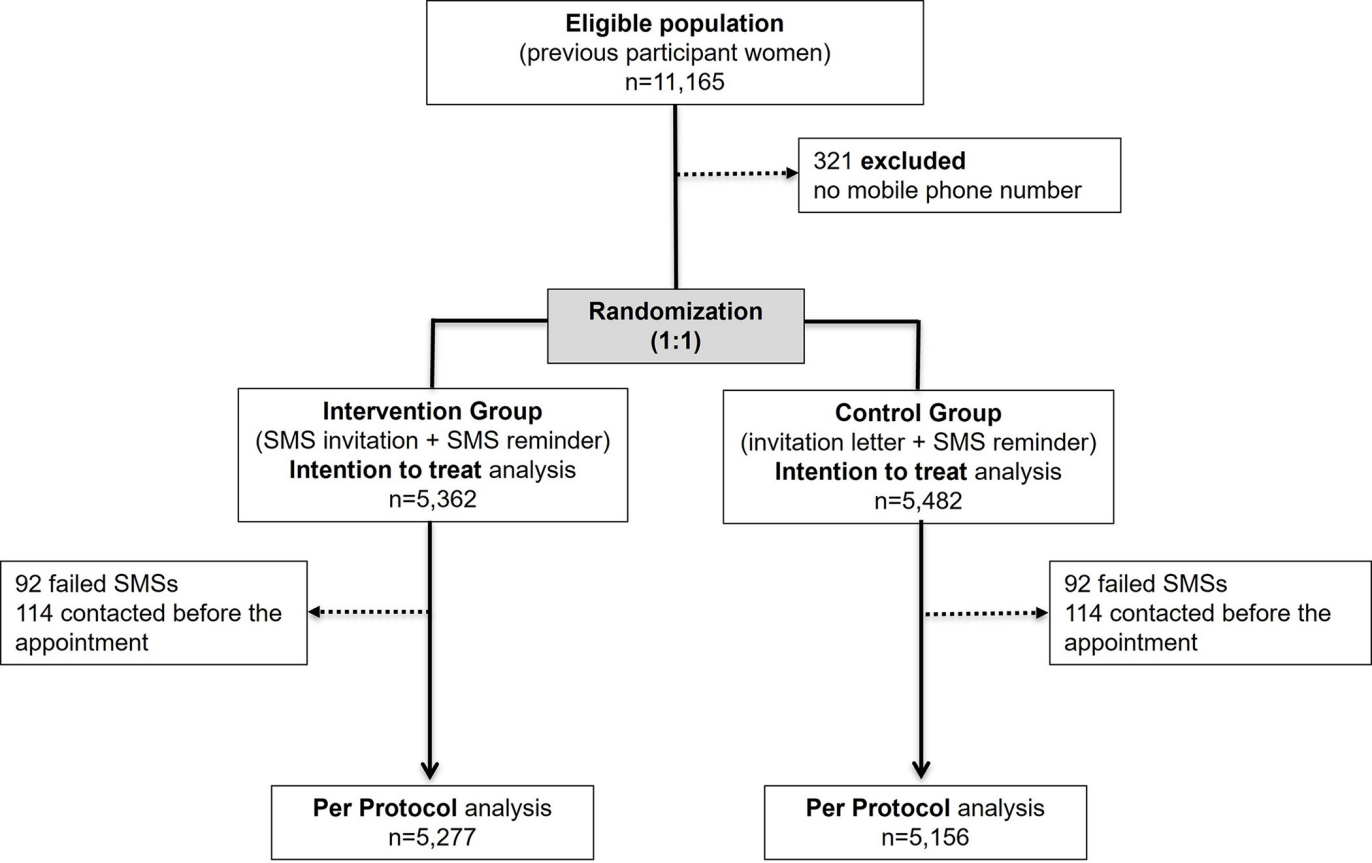
CONSORT flow diagram of invitation intervention to previous participant women in a breast cancer screening program.

Between 14 September 2021 and 26 April 2022, 11,165 women were enrolled in this study. We excluded 321 women (2.9%) with no mobile phone number registered. Of the 10,844 women included, 5,482 were randomly assigned to the control group and 5,362 to the text message invitation group. Text messages failed to be delivered in 92 (1.7%) women assigned to the intervention group; therefore, invitation letters were sent. Letters failed to be delivered in 72 (1.3%) women assigned to the control group. The intention-to-treat analysis included 10,844 participants, and the per-protocol analysis included 10,433 participants (Fig 1).

The mean age of women recruited was 60.3 years (SD 5.3), with 3,732 (34.4%) from a high DS area. Baseline characteristics were balanced across the two groups (Table 1).

**Table 1:** Baseline characteristics of the study subjects by trial group.

	Text-message	Letter	* *	Total
	n (%)	n (%)	*P value*	n (%)
Age, years				
Mean (SD)	60.3 (5.3)	60.4 (5.3)	*0*.*249*	60.3 (5.3)
Age groups, years				
52–54	1,361 (25.4)	1,339 (24.4)	*0*.*514*	2,700 (24.9)
55–59	1,174 (21.9)	1,175 (21.4)	2,349 (21.7)
60–64	1.626 (30.3)	1,711 (31.2)	3,337 (30.8)
65–69	1,201 (22.4)	1,257 (22.9)	2,458 (22.7)
Deprivation Score				
1st tertile	1,288 (24.0)	1,284 (23.4)	*0*.*764*	2,572 (23.7)
2nd tertile	2,236 (41.7)	2,304 (42.0)	4,540 (41.9)
3rd tertile	1,838 (34.3)	1,894 (34.5)	3,732 (34.4)
**Total**	**5,362**	**5,482 **	** **	**10,844**

### Primary outcome

**Fig 2:**
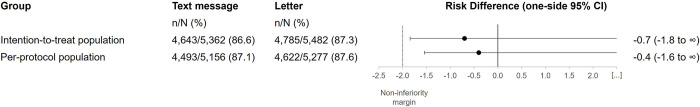
Differences in participation at 12 weeks after invitation according to the analysis performed.

The screening participation rate within 12 weeks of the invitation was 86.6% in the text message group and 87.3% in the letter group. The difference in the participation rate in the intention-to-treat population was -0.7 percentage points (95% one-side confidence interval [CI], −1.8 to ∞), thus meeting the noninferiority criteria based on the 2-percentage-point margin. The per-protocol analysis was consistent with the intention-to-treat analysis (Fig 2). Subgroup analyses by age and DS index consistently showed no differences in participation rate among the two groups (*P>0*.*05)* (Table 2).

**Table 2:** Subgroup analysis of participation rate at 12 weeks after invitation by trial arm (intention-to-treat).

	Letter invitation* n/N (%)	Text-message invitation n/N (%)	OR (95%CI)	*P value*
Age groups, years				
52–59	2153/2514 (85.6)	2146/2535 (84.7)	0.93 (0.79–1.08)	*0*.*333*
60–69	2632/2968 (88.7)	2497/2827 (88.3)	0.96 (0.82–1.13)	*0*.*658*
Deprivation Score				
1st tertile	1132/1284 (88.2)	1104/1288 (85.7)	0.80 (0.63–1.01)	*0*.*057*
2nd tertile	1979/2304 (85.9)	1938/2236 (86.7)	1.08 (0.91–1.28)	*0*.*381*
3rd tertile	1674/1894 (88.4)	1601/1838 (87.1)	0.89 (0.73–1.08)	*0*.*251*

* Reference group (adjusted for age and Deprivation Score)

In the intervention group, there was no difference in participation between women who received one text message as the invitation and those who received two consecutive text messages (notification plus invitation) incorporated after interim analysis (87.9% vs. 86.7%, respectively, P = 0.212). (S2 Table).

### Secondary outcomes

Table 3 summarizes secondary outcome measures. Attendance to the initial timed appointment was higher among women who received the invitation by text message compared to those who received the invitation by letter (87.7% vs 85.3%, P<0.002). However, cancellation of the initial appointment was higher among women invited by letter than by text message (21.1% vs. 15.1%, respectively, P<0.007).

**Table 3:** Timing of screening attendance (initial vs. rescheduled appointment) and cancellation rate by trial arm *.

	Text message (4,729)	Letter (4,626)	* *
	n (%)	n (%)	*P value*
**Attendance**	4,024	4,133	
Attendance to initial appointment	3,529 (87.7)	3,526 (85.3)	*0*.*002*
Attendance after rescheduling initial appointment	495 (12.3)	607 (14.7)	
**No attendance**	602	596	
Cancellation before initial appointment	91 (15.1)	126 (21.1)	*0*.*007*
No cancellation	511 (84.9)	470 (78.9)	* *

*Out of the total women recruited, 1,489 were excluded from this analysis due to the complexity of the mammography schedules, which made it impossible to calculate these outcomes accurately (753 from the text message arm and 736 from the letter arm).

## Discussion

This randomized trial assessed the use of text messages as an alternative invitation method to mammographic screening in women with previous participation in a BC screening program. Our study did not find significant differences in participation rates, which suggests that text messages may be an alternative invitation method to traditional invitation letters for mammographic screening in subsequent invitations to previously participating women in a BC screening program. We also observed slightly higher attendance at the initial appointment and lower rescheduling and cancelation rates for text message invitations compared to letters.

Previous studies have shown the effectiveness of text messages as invitation reminders to increase participation in BC screening programs [12, 20]. However, as far as we know, text messages have not been previously assessed as the primary method for inviting women to participate in BC screening.

The long-term effectiveness of BC screening programs is closely related to high adherence among the target population. Detecting BC early is not ensured by one-off screening participation but by the consistency of participation in line with recommended time intervals [21]. The literature suggests that women who have had previous mammograms are more likely to continue to have regular screenings. This is because of a belief in the effectiveness of screening, which increases their intentions to go for screening and results in their adherence to subsequent screens [22]. In this sense, our study shows that adherence to mammography screening was considerably high, above 86.9%, with no significant difference between the text message group and the letter group (86.6% and 87.3%, respectively). However, efforts to improve BC screening programs are necessary even with high participation rates for better public health outcomes.

To optimize the workflow within the screening units, we use a scheduled model based on estimates of users’ attendance probabilities. Our screening program invitations include a fixed mammography appointment as a strategy to ensure adequate participation. This reduces organizational barriers and the need for women to contact the screening hub to request a mammography appointment [23, 24]. We found that women who received the invitation by text message attended more at the initial timed appointment than those who received the invitation by letter, probably because it was received without delay, allowing them to better adjust their agendas. However, text message invitations did not improve cancellation rates as expected. As cancellation or rescheduling rates can be influenced by factors related to appointment systems that make it difficult to cancel or reschedule (e.g., busy phone lines), it is crucial to provide as easy and simple a system as possible for people who have been invited to be screened to respond and to improve the efficiency of these programs [14].

The present study has some limitations. Firstly, determining the margin of noninferiority (clinical relevance) was challenging. To address this issue, we established the margin of noninferiority by considering the participation variability of women in this group over the last decade. According to previous studies, a small increase in participation can lead to a significant reduction in advanced stage and death from BC [25]. Secondly, while we were able to confirm the delivery of the text message, we could not determine whether the recipient had read and understood its content. Nonetheless, this limitation was applicable to the invitation letters as well, and it was anticipated to impact both groups similarly.

Opting for text messages instead of invitation letters can reduce the cost of inviting women with previous participation in BC screening due to the substantial difference in costs between text messages (€0.05) and postal letters (€0.51). This cost reduction could be used to implement tailored strategies that encourage engagement among non-attenders, resulting in better outcomes and more efficient resource allocation. In this regard, Robotham et al. conducted a systematic review aimed at investigating the impact of multiple notifications on participation rates. The researchers found that two or more notifications increased participation by as much as 19% over and above sending one notification [26].

Our study was addressed to previously participating women in the BC screening program who had previously been informed of the benefits and risks, having opted to participate. To potentially use text messages as an invitation method to the entire target population, it will be necessary to include communication on benefits and risks in the message, for example, by including hyperlinks in the text message to web tools to help the decision-making process. However, security and mistrust of text messaging can limit its role for first-time invitees. When individuals receive a message from a health service that they never interacted with before, they may lack confidence in the message’s authenticity and content. To establish trust, it is recommended to be consistent across different communication channels, such as publishing contact details and links on websites and in letters so that individuals can check the identity of the sender and the authenticity of the message [27].

Mobile phone numbers of women in our screening program were widely available, therefore, using mobile devices for mammography scheduling and reminders not only encourages women to undergo regular screenings but also enhances the efficiency of healthcare systems by reducing the likelihood of missed appointments. Mobile scheduling and reminder systems can provide real-time updates to users in case of unexpected changes in screening unit schedules, ensuring that they are informed in a timely manner [28]. Integrating mobile solutions into broader healthcare systems would also ensure effective follow-up and treatment for individuals with positive screening results, such as My Health digital personal space [14, 29, 30].

## Conclusions

This randomized controlled trial provides valuable insights into the use of alternative invitation methods for population-based cancer screening programs. A text message invitation for women who had previously participated in breast cancer screening was not inferior to the standard letter. In addition, it would reduce costs and contribute to environmental sustainability. Further research is needed to determine the best timing and frequency of text messages for better outcomes and identify strategies for facilitating rescheduling or cancellation.

## Supporting information

S1 Checklist CONSORT 2010 checklist of information to include when reporting a randomised trial*. (DOC)

S1 File Content of the text message and letter invitation. (DOCX)

S1 Table Participation rate at 12 weeks by trial group in the interim analysis. (DOCX)

S2 Table Participation rate at 12 weeks in the intervention group comparing the use of one or two text message invitation. (DOCX)
