# Supplementary material for: The use of text messages as an alternative invitation method for breast cancer screening: A randomized controlled trial (M-TICS study)
Source: PLoS One. 2024 Aug 29;19(8):e0306720. doi: 10.1371/journal.pone.0306720 (PMC11361687; doi:10.1371/journal.pone.0306720)
Supplement: S1 File — (DOCX) [file pone.0306720.s002.docx]

**Standard procedure (control group) in our breast cancer screening program**

- **Invitation letter sent to previous participating women**


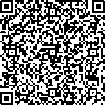
Name and Surname

Postal address

Zip code

# BC screening code number

Online information leaflet

Dear madam,

Following the recommendation of the results from your previous screening mammography, we invite you again to participate in the Breast Cancer Early Detection Program.

**This is your appointment:**

Date: **dd/mm/yyyy** at **hh:mm** Name of the **Center**

Postal address of the center

Remember to bring your Health Card

Once your screening mammography has been performed, you will receive a letter with your results. In most cases, there is no need to repeat the exam for up to 2 years. In case you need to perform further study, we will contact you to give you a new appointment and perform the appropriate complementary examinations.

# Please contact us:

- If you are unable to attend or wish to reschedule your appointment
- If you have had a mammography in the past 6 months.
- If you are being screened outside of our Program
- **If you have had breast cancer**

**Telephones (Monday through Friday, from 9:00 AM to 3:00 PM)**

**Web:** [**https://www.icoprevencio.cat/mamografia/cat**](https://www.icoprevencio.cat/mamografia/cat)

**E-mail:** [**prevenciomama@iconcologia.net**](mailto:prevenciomama@iconcologia.net)

If your mobile number is available, we will send you an SMS reminder a few days before the appointment, so that you can text back if you need to cancel or reschedule your appointment.

Kindly,

Name of the coordinator of the Breast Cancer Screening Program.

The Catalan Institute of Oncology, in compliance with Regulation (EU) 2016/679 of the European Parliament and of the Council of 27 April 2016 on the protection of individuals with regard to the processing of personal data and on the free movement of such data, informs you that the processing of your data will be to ensure the registration, management, and monitoring of the information arising from your participation in the program, as well as for health research purposes always in an anonymized form. This information will be used by the administrative services and services directly linked to the health care of our entity, each in its competencies, and may be sent in whole or in part to public and private official bodies which, for legal reasons or for reasons of material necessity, must have access to the data for the purposes of the correct provision of the medical-health care that constitutes the purpose of the processing of these data. The data provided will be kept in accordance with the health legislation in force at any given time. You have the right to exercise your rights of access, rectification, deletion, limitation of processing, portability, and opposition of your data by writing to the Data Protection Officer at lopd@iconcologia.net, in any case, you must attach a photocopy of your national identity card or equivalent. Likewise, you are informed of your right to, if you do not agree with the treatment carried out by our entity or consider your rights have been violated, file a complaint at any time before the Catalan Data Protection Authority.

Responsible for the treatment: Catalan Institute of Oncology | CIF: Q5856383D | Postal address: Av. Gran Via, 199-203. 08908 Hospitalet de Llobregat | E-mail: lopd@iconcologia.net | Data Protection Coordinator: [lopd@iconcologia.net](mailto:lopd@iconcologia.net)

-
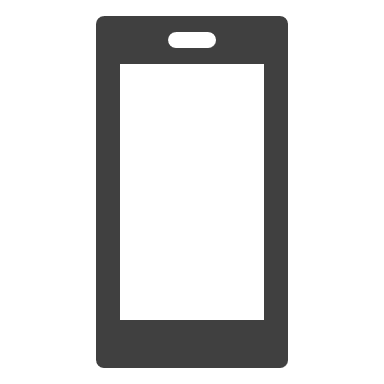
**Text message reminder sent 3 days before the appointment (if mobile phone number registered)**

ICO reminds your MAMOGRAPHY appointment at the Center on dd/mm/yyyy hh:mm. Reply to this message if you need to reschedule or cancel.

**Intervention procedure**

- **Text message invitation sent to previously participating women**
  -
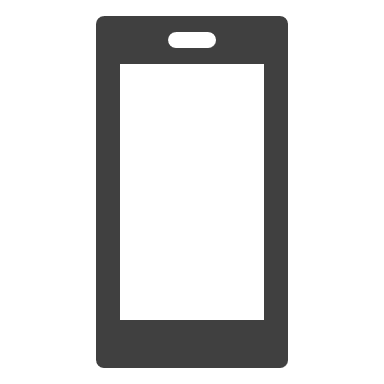
Unic text message invitation (first part of the study):

X SURNAME, ICO Cancer Prevention has scheduled your appointment for a routine MAMMOGRAPHY at the Center on dd/mm/yyyy hh:mm

Information: <https://t.ly/kRcy>

- - Two consecutive text message invitations (second part of the study):

X SURNAME, ICO Cancer Prevention has scheduled your appointment for a routine MAMMOGRAPHY at the Center on dd/mm/yyyy hh:mm. Information: <https://t.ly/kRcy>


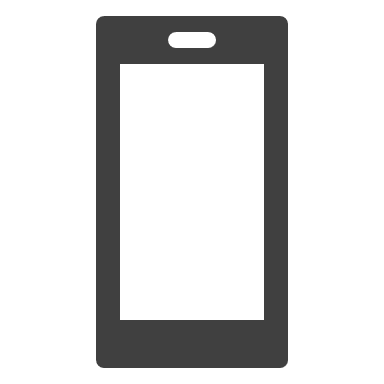


X SURNAME, ICO Cancer Prevention will send you an SMS invitation to get a screening mammography. This message replaces the usual letter you used to receive every 2 years.


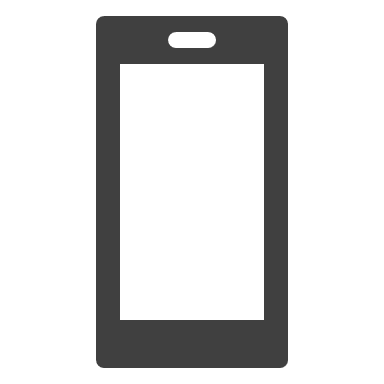


- **Content of the text message link:**

This message replaces the biennial letter invitation with a mammography appointment for early detection of breast cancer.

Here is the center and address information you need.

Name of the **Center**

Postal address of the center

# Please contact us:

- If you are unable to attend or wish to reschedule your appointment
- If you have had a mammography in the past 6 months.
- If you are being screened outside of our Program
- **If you have had breast cancer**

**Telephones (Monday through Friday, from 9:00 AM to 3:00 PM)**

**Web:** [**https://www.icoprevencio.cat/mamografia/cat**](https://www.icoprevencio.cat/mamografia/cat)

**E-mail:** [**prevenciomama@iconcologia.net**](mailto:prevenciomama@iconcologia.net)

Kindly,

Name of the coordinator of the Breast Cancer Screening Program.

The Catalan Institute of Oncology, in compliance with Regulation (EU) 2016/679 of the European Parliament and of the Council of 27 April 2016 on the protection of individuals with regard to the processing of personal data and on the free movement of such data, informs you that the processing of your data will be to ensure the registration, management, and monitoring of the information arising from your participation in the program, as well as for health research purposes always in an anonymized form. This information will be used by the administrative services and services directly linked to the health care of our entity, each in its competencies, and may be sent in whole or in part to public and private official bodies which, for legal reasons or for reasons of material necessity, must have access to the data for the purposes of the correct provision of the medical-health care that constitutes the purpose of the processing of these data. The data provided will be kept in accordance with the health legislation in force at any given time. You have the right to exercise your rights of access, rectification, deletion, limitation of processing, portability, and opposition of your data by writing to the Data Protection Officer at lopd@iconcologia.net, in any case, you must attach a photocopy of your national identity card or equivalent. Likewise, you are informed of your right to, if you do not agree with the treatment carried out by our entity or consider your rights have been violated, file a complaint at any time before the Catalan Data Protection Authority.

Responsible for the treatment: Catalan Institute of Oncology | CIF: Q5856383D | Postal address: Av. Gran Via, 199-203. 08908 Hospitalet de Llobregat | E-mail: lopd@iconcologia.net | Data Protection Coordinator: [lopd@iconcologia.net](mailto:lopd@iconcologia.net)

-
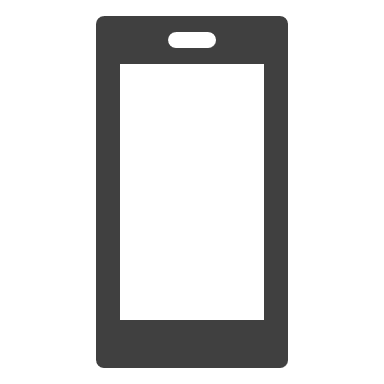
**Text message reminder sent 3 days before the appointment (if mobile phone number registered)**

ICO reminds your MAMOGRAPHY appointment at the Center on dd/mm/yyyy hh:mm. Reply to this message if you need to reschedule or cancel.
