# Supplementary material for: The use of text messages as an alternative invitation method for breast cancer screening: A randomized controlled trial (M-TICS study)
Source: PLoS One. 2024 Aug 29;19(8):e0306720. doi: 10.1371/journal.pone.0306720 (PMC11361687; doi:10.1371/journal.pone.0306720)
Supplement: S1 Table — (DOCX) [file pone.0306720.s003.docx]

**Supplementary table 1. Participation rate at 12 weeks by trial group in the interim analysis.**

|  | **Text message** |  | **Letter** |  | ***P value*** |
| --- | --- | --- | --- | --- | --- |
|  | n/N (%) |  | n/N (%) |  |  |
| Intention-to-treat population | 1,696/1,942 (87.3) | | 1,779/2,010 (88.5) | | *0.257* |
| Per-protocol population | 1,577/1,792 (88.0) |  | 1,656/1,863 (88.9) | | *0.402* |
