# Supplementary material for: The use of text messages as an alternative invitation method for breast cancer screening: A randomized controlled trial (M-TICS study)
Source: PLoS One. 2024 Aug 29;19(8):e0306720. doi: 10.1371/journal.pone.0306720 (PMC11361687; doi:10.1371/journal.pone.0306720)
Supplement: S2 Table — (DOCX) [file pone.0306720.s004.docx]

**Supplementary table 2. Participation rate at 12 weeks in the intervention group comparing the use of one or two text message invitation**

|  | **One text message**  **invitation** |  | **Two text message**  **invitation** |  | ***P-value*** |
| --- | --- | --- | --- | --- | --- |
|  | n/N (%) |  | n/N (%) |  |  |
| Intention-to-treat population | 1,675/1,906 (87.9) | | 2,916/3,364 (86.7) | | *0.212* |
| Per-protocol population | 1,577/1,792 (88.0) |  | 2,916/3,364 (86.7) | | *0.178* |
